# Supplementary material for: The impact of mixed-ownership reform on zombie firms: Evidence from Chinese listed SOEs
Source: PLoS One. 2024 Jul 3;19(7):e0301048. doi: 10.1371/journal.pone.0301048 (PMC11221639; doi:10.1371/journal.pone.0301048)
Supplement: Attachment — Submitted filename: Response to Reviewers.docx [file pone.0301048.s002.docx]

Dear editor,

Thank you for your valuable comments on this paper. It is very helpful for us to recognize the problems existing in the paper and make the article more rigorous and reasonable. We would like to thank the editor and reviews for their questions and suggestions. We have made a revision to the article, including abstract, introduction, empirical model, robust test, and policy recommendation.

We have revised and replied to the questions raised by the experts one by one, which are attached below, thank you again! Now is the Chinese New Year holiday, the best wishes for you!

Looking forward to your reply, thank you very much!

Sincerely yours

**Additional Editor Comments:**

**1.** Given your research focus on China, you need to spend some efforts to explain (1) why the Chinese context is particularly relevant to your research question.

**Answer: Thank you for your suggestions.** Choosing China as our research object is based on the following considerations: clearing out zombie companies is a challenge for both developed and developing countries. Unlike zombie firms in developed countries, Zombie firms in China have typical characteristics of government intervention. At the same time, China, as one of the world's largest economies, is currently undergoing a critical period of economic transformation, marked by the implementation of policies such as mixed ownership reform. Studying the impact of mixed ownership reform on zombie firms holds significant importance for both developing and developed countries. Different from previous literature on using external macroeconomic policy adjustments to clear out zombie firms, this article may provide a way to clear out zombie firms from the perspective of mixed-ownership reform within enterprises.

**2.** whether the results can be generalized to other empirical settings. You may want to consider the following studies that offer different approaches to the determinants or conditions of zombie firms: <https://ssrn.com/abstract=3829038>, <https://doi.org/10.7441/joc.2023.02.05>,

<https://doi.org/10.1016/j.japwor.2023.101188>.

**Answer: Thank you for providing us with the references, which are very helpful to us.** We believe the results in the article can be generalized to other empirical settings. The key point to clear out zombie firms lies in enhancing their competitiveness. There are two issues that need to be solved: one is who is a zombie firm, and the other is how to effectively clean up zombie firms. Firstly, this article proposes an improved identification standard of zombie firms based on the FN-CHK standard, which helps to improve the accuracy of identifying zombie firms. Secondly, this article confirms that the participation of non-state-owned shareholders in the corporate governance of state-owned enterprises can reduce government intervention, strengthen the competitiveness of enterprises, and thus help to clear out zombie firms. The conclusion of this article has significant implications for the clearing out of zombie firms in developed and developing countries.

The firs literature (<https://ssrn.com/abstract=3829038>) builds the RDT model to examinate of the risks taken on by a company during the insolvency process. We believe that it makes a useful supplement to the conclusions of this article. Because not all zombie firms need to go bankrupt, some firms can be revived through some rescue measures (such as restructuring). The first literature helps to determine whether a firm is restructuring or going bankrupt. The conclusion of this article is helpful for how to restructure and leverage the advantages of heterogeneous shareholders to enhance the competitiveness. The second literature (<https://doi.org/10.7441/joc.2023.02.05>), soft constraint constraints are an important cause of zombie firms in Japan's manufacturing industry. The conclusions of this article show that mixed-ownership reform helps to breaking soft constraint constraints, which is beneficial for clearing out zombie firms. In the third literature (<https://doi.org/10.1016/j.japwor.2023.101188>), a firm is defined as a zombie firm with an interest coverage ratio of below one over the previous three years. The above literature has provided a foundation for identifying firms. However, it must be acknowledged that due to differences in financial system structure and financial environment, the identification standards for zombie firms still need to be continuously improved. This is a direction that needs to be studied on in the future.

**3.** In the caption of Table 2 and subsequent please explain the significance of numbers in paratheses;

**Answer: Thank you for your suggestions.** We have added the explanation for the significance of numbers in paratheses in the caption of Table 2 and subsequent.

**4.** Insert reference to the limitations of the study before the conclusion section;

**Answer: Thank you for your suggestions.** We have added the limitations of the study and the future research directions as follows:

Although this article provides a comprehensive examination of the relationship between mixed ownership reform and zombie firms, there are still some limitations and directions for further exploration. Firstly, it's important to acknowledge that this article's focus on state-owned enterprises as research samples may introduce bias due to the exclusion of non-listed state-owned enterprises. This limitation could influence regression results and potentially skew conclusions. Additionally, the absence of consideration for the impact of COVID-19 on zombie firms suggests a potential subjectivity in the research findings. Therefore, we encourage future studies to broaden their scope by incorporating data from both developing and developed economies over longer temporal and spatial spans to provide a more comprehensive analysis. Secondly, while the mixed ownership reform entails the integration of state-owned and non-state-owned capital, future research could explore the effects of state-owned capital's involvement in the corporate governance of private enterprises, particularly in addressing issues related to private zombie firms. Lastly, bankruptcy represents a significant mechanism for clearing out zombie firms. Further research could delve into strategies for mitigating the employment and social stability challenges that accompany the bankruptcy of zombie firms.

**5.** Please double check the English writing, there are some errors in the text.

**Answer: Thank you for your suggestions.** We have carefully checked and corrected the errors and inappropriate expressions in the paper. In future research, we will pay special attention to this point. **Thank you again.**

**Reviewer #1**

**1.** In my opinion, I would reconsider the link between the proposed hypothesis and the model from eq. (1) (also where you defined the dependent variable). Your proposed hypothesis states that: Under certain other conditions, mixed-ownership reform can promote the governance of zombie firms. So, in these conditions the governance of zombies would be the dependent variable, and from the eq (1) the dependent variable is Zombie (a composite score variable - I guess) that describe a status based on Pre-tax net profits, Asset-liability ratios and Yearly loan amounts. Based on these, I don't find a link between this status and governance. Please try to answer this question: how do you measure governance of zombie firms and what is the link between those metrics and the Zombie variable.

**Answer: Thank you for your suggestions very much!** We have reconsidered the meaning of the term "governance" and found that it does not accurately match the content of this article. The variable of "zombie firm" in this article is a 0-1 variable. The empirical regression results of this article show that the regression coefficient of mixed-ownership reform on zombie firms is significantly negative, and mixed-ownership reform helps to improve the efficiency of state-owned enterprises, thereby reducing the possibility of SOEs becoming zombie firms. In practical scenarios, a cohort of zombie firms such as Shandong Yanmei International Coking and Youyi Special Steel have embraced a union of "state-owned framework and private mechanisms" through mixed-ownership reform, igniting the resource integration capabilities of SOEs and invigorating market mechanisms within private enterprises. For instance, Shandong Yanmei International Coking achieved a notable milestone in the year of mixed-ownership reform (2020) by realizing a total profit of 502 million yuan, signifying a staggering 12% year-on-year growth, while concurrently reducing its debt-to-asset ratio from 104% in 2017 to 61.54% in 2020. Therefore, this article uses the term "governance" to show the positive effect of mixed-ownership reform on zombie firms. However, based on your suggestion, we believe that a more accurate expression of the hypothesis in this article should be that " Under certain other conditions, mixed-ownership reform helps to reduce the possibility of state-owned enterprises (SOEs) becoming zombie firms". At the same time, we have also made corresponding modifications in the article. **Thank you again!**

**2.** Please motivate the sample period selection: why 2019 and not the current year.

**Answer: Thank you for your question!** The selection of sample period is mainly based on the following two considerations: (1) After 2019 means COVID-19. It causes enormous harm to the global economy, and the Chinese economy is no exception. The slowdown in macroeconomic growth may give rise to more zombie firms. After the COVID-19, it is still necessary to wait for enough time to test whether zombie firms that have carried out mixed-ownership reform before the COVID-19 or during the COVID-19 have stronger recovery capacity. At present, the impact of the COVID-19 epidemic on zombie firms is rare in the existing literature, and it is challenging to accurately assess the resilience of zombie firms brought about by the mixed-ownership reform, which is also a complex problem that is worth continuing to study. We also state this in the limitations of this article. (2) This article measures the degree of non-state-owned shareholder participation in the mixed-ownership reform from equity structure and board structure. This requires manual collection and identification of information on the top ten shareholders from existing databases, including key data such as shareholding ratios and shareholder nature. Due to difficulties in data, our team has only collected data before 2019, and we are working hard on data after 2019. **Thank you again!**

**Reviewer #2:**

**1.** Authors must use the impersonal style (to give up expressions like ...our study...we...etc.).

**Answer:** **Thank you for your suggestions.** We have changed the expressions like …our study…we…etc into the impersonal style. In future writing, we will also pay special attention to this point. Thank you!

2. The authors must present, in the introduction, the existing situation worldwide and briefly present the European experience.

**Answer: Thank you for your suggestions.** We have added the existing situation worldwide and the European experience in the second paragraph of the introduction section. This section is necessary as it contributes to the coherence of the article. In future writing, we will pay attention to this point. The modified content is as follows:

The study of zombie firms originated from an examination of Japan's "lost decade" in the 1990s. The earliest academic research that defined zombie firms was conducted by Hoshi (2006) and Caballero et al. (2008). Zombie firms refer to businesses that are no longer profitable and can only survive with assistance from banks. In the case of Japan, with the deterioration of the economy, many Japanese companies were unable to even pay the interest on their debts. Under normal circumstances, these firms would have gone bankrupt. However, to avoid admitting to their shareholders that the loans were unlikely to be fully repaid, many banks allowed these struggling firms to suspend their payments. As a result, the banking industry kept these financially distressed firms afloat for an extended period of time. Zombie firms are not limited to Japan; they are prevalent in both developed and developing countries worldwide. Extensive research conducted by Adalet McGowan et al. (2018) has revealed a significant increase in the number of zombie firms across advanced economies following the Global Financial Crisis (GFC). Similarly, Banerjee and Hofmann (2022) found a substantial rise in zombie firms, with their prevalence increasing from approximately 4% of all listed firms in the mid-1980s to as high as 15% in 2017 across 14 OECD countries. In Europe, the issue of zombie firms became particularly pronounced after the GFC and the subsequent European sovereign debt crisis. During this period, unconventional monetary policies led to weakly capitalized banks extending loans to struggling firms, a practice known as "evergreening" (Acharya et al., 2019). The introduction of Very Long-Term Refinancing Operations (VLTROs) further exacerbated the problem, as zombie firms obtaining new and larger loans from banks faced higher expected default probabilities (Christian et al., 2021). In the context of the COVID-19 and slowdown in the global economy, central banks in various countries have implemented monetary easing policies, deepening the dependence of more enterprises on debt. The increasing number of zombie firms in the Eurozone and other countries has emerged as an urgent issue that requires immediate attention and resolution.

3. At the end of the literature review section, the authors must insist on identifying a research gap that justifies the completion of this study.

**Answer: Thank you for your suggestions.** We have added the research gap in the literature review section as follows:

While there is a sufficient literature on zombie firms and mixed ownership reform, there exist some gaps in the existing research. This article fills research gaps in two ways: (1) Effectively addressing the issue of zombie firms is a critical challenge for both developed and developing countries worldwide. This article presents research findings indicating that the introduction of non-state-owned shareholders through mixed ownership reform has a positive impact on resolving zombie firms. Compared to adopting "external" economic policies to clear out zombie firms, this article provides evidence for clearing out zombie enterprises from the perspective of "within the enterprise" and supplements the literature on clearing out zombie firms. (2) Unlike previous studies on developed economies, this article focuses on Chinese listed SOEs and empirically examines the impact of mixed ownership reform in transitional economies on zombie firms from the perspectives of equity structure and board governance. This has a profound impact on promoting sustainable and healthy economic development.

4. The authors must justify in the paper the choice of the country.

**Answer: Thank you for your suggestions.** We have added the discussion about the choice of the country in “3.3 Date source”. Your suggestions have helped us improve the comprehensiveness of the article. Thank you again! The revised content is as follows:

The reasons for selecting Chinese listed SOEs as the research sample are as follows: (1) China, as one of the world's largest economies, is currently undergoing a critical period of economic transformation, marked by the implementation of policies such as mixed ownership reform. Studying the impact of mixed ownership reform on zombie firms holds significant importance for both developing and developed countries to eliminate zombie firms, and foster sustainable and healthy economic development.

5. The authors must insert a discussions section. In the discussion section, the authors must interpret the results obtained in the context of similar studies that confirm or refute their results.

**Answer: Thank you for your suggestions.** We have inserted a discussion in the paper. The discussion section is very useful for making the paper more comprehensive. The modified content is as follows:

The results obtained in the previous article are mainly discussed in this section. Firstly, the results show that the coefficient of mixed-ownership reform to zombie firms is significantly negative, indicating that mixed-ownership reform can help reduce the possibility of state-owned enterprises (SOEs) becoming zombie firms. In reality, the issue of addressing zombie firms is of paramount importance for both developed and developing economies. However, related literature remains relatively limited. Existing studies have explored the ways to address zombie firms through several lenses, including relationship banking[60], bank competition[61], FinTech[62], digital transformation [63] and The Belt and Road Initiative[64]. Moreover, the research conducted by Ernesto et al. (2022) [65]offers valuable insights into the effectiveness of institutional reforms in Portugal, an OECD country grappling with a high prevalence of zombie firms. Contrary to conventional wisdom, the findings suggest that not all zombie firms are inherently doomed and the right institutional framework in place can facilitate their restructuring and eventual recovery, which could be more beneficial than simply forcing them into bankruptcy. Furthermore, Fischer (2021)[60] and Carreira et al. (2022)[66] support these findings by highlighting the positive impact of restructuring strategies in preventing the emergence of zombie firms and facilitating their revitalization. Given this, will the heterogeneous shareholder equity integration and resource integration brought about by mixed-ownership reform help clear out zombie firms? As a developing country in transition, China's SOEs play a crucial role in economic development, but at the same time, the risk of evolving into zombie firms is also higher[67]. Based on this, this article takes Chinese listed SOEs as the object and confirms the positive impact of mixed ownership reform on reducing the risk of zombification in SOEs, thus offering valuable theoretical insights into clear out zombie firms.

Secondly, research by Leire et al. (2022)[68] and Veganzones and Severin (2023)[69] has demonstrated that enhancing corporate governance can mitigate the prevalence of zombie firms. However, this article extends "corporate governance" to " corporate governance of mixed-ownership enterprises". Focusing on Chinese listed State-Owned Enterprises (SOEs), the study evaluates mixed-ownership reform by assessing the proportion of non-state shareholders appointing directors (supervisors and executives) relative to all directors. The findings indicate that non-state shareholders' direct involvement in appointing directors to SOEs significantly diminishes the zombification risk of SOEs, whereas mere participation in shareholding does not yield the same effect. The appointment of directors by non-state-owned shareholders significantly enhances the diversity of SOEs' boards, reduces the prevalence of dual roles, decreases government entanglement, and effectively bolsters governance efficiency. Moreover, it alleviates policy burdens on SOEs and diminishes the likelihood of them becoming zombie firms.

Thirdly, the findings suggest that the reduction of inefficient investment plays a crucial mediating role in the relationship between mixed-ownership reform and zombie firms. Previous studies have highlighted the propensity of Chinese SOEs to experience heightened government intervention, coupled with subsidies, biased industrial policies, and local government protection. These factors often contribute to excessive investment and diminished investment efficiency[53]. This article introduces inefficient investment as a mediator variable to delve deeper into the relationship"mixed-ownership reform, inefficient investment, and zombie firms", thereby enriching existing theoretical frameworks. The observed results may stem from the pronounced profit orientation of non-state-owned shareholders, who actively engage in the governance of SOEs, thereby enhancing investment efficiency and reducing the likelihood of SOEs transitioning into zombie firms. In addition, different from prior research conducted on large and medium-sized enterprises in Germany[60] and small and medium-sized enterprises in Portugal[66], this article chooses SOEs (mainly medium-sized and large enterprises) as a sample and has value for existing literature. Moreover, the findings also underscore the heightened impact of mixed-ownership reform within competitive industries and the eastern regions of China. This discovery holds substantial policy implications, highlighting the absence of a one-size-fits-all solution for addressing zombie firms. Instead, a tailored approach akin to targeted drugs is imperative for effectively tackling this issue.

6. More additional references can be used in the introduction, LR, discussions sections of the paper Blažková, I., & Chmelíková, G. (2022). Zombie Firms during and after Crisis. Journal of Risk and Financial Management, 15(7), 301.; Rodano, G., & Sette, E. (2019). Zombie firms in Italy: a critical assessment. Bank of Italy Occasional Paper, (483).; Banerjee, R., & Hofmann, B. (2022). Corporate zombies: Anatomy and life cycle. Economic Policy, 37(112), 757-803.; El Ghoul, Sadok, Zhengwei, Fu, Guedhami, Omran. (2021). Zombie firms: Prevalence, determinants, and corporate policies. Finance Research Letters, 41, 101876.

**Answer: Thank you for your suggestions.** The references you provided are very helpful, and we greatly appreciate them! The references have been used in our article.

7. The authors must improve the conclusions section taking in account the quality of the study and extend the policy implications section.

**Answer:** **Thank you for your suggestions.** Your suggestions are very useful to us. We have revised the research findings and policy implications as follows:

The Chinese economy is undergoing a pivotal phase of structural transformation, wherein the presence of zombie firms poses a significant obstacle to its health and long-term sustainability. Examining the effect of mixed ownership reform on zombie firms holds paramount importance in identifying strategies to eradicate them and foster a path towards enduring economic development. Based on data from Chinese listed SOEs, this article examines the effect of mixed-ownership reform on zombie firms, yielding the following conclusions: Firstly, mixed-ownership reform contributes to clearing out of zombie firms. Directors appointed by non-state-owned shareholders play a crucial role in significantly diminishing the occurrence of zombie enterprises. Conversely, non-state-owned shareholders who solely engage in shareholding do not yield the same effect in addressing this issue. This conclusion remained robust after a series of rigorous tests. Secondly, mixed-ownership reform exhibits a more significant inhibitory effect on zombie firms in competitive industries and the eastern region. Thirdly, inefficient investment plays an intermediary role between mixed-ownership reform and zombie firms. This means that mixed ownership reform reduces the likelihood of zombie firms by reducing inefficient investment, which is beneficial for clearing out zombie firms.

Based on the conclusions of this study, several policy recommendations are discussed as follows. Firstly, emphasizing a market-driven approach is crucial for effectively promoting mixed-ownership reform. Mere amalgamation of state-owned and private ownership is insufficient; it is imperative to actively engage non-state-owned shareholders in the governance of State-Owned Enterprises (SOEs), empowering them with greater influence to fully realize the policy objectives of mixed-ownership reform. Secondly, clearing out zombie companies is a global challenge: providing assistance to firms on the verge of bankruptcy can inadvertently perpetuate their zombie status. Conversely, withholding assistance may deny potentially viable businesses the chance to recover. Furthermore, research indicates that even recovered zombie firms tend to exhibit weak resilience and are susceptible to relapse into zombie status (Banerjee and Hofmann, 2022). The conclusion of this article indicates that market-oriented and legal measures should be adopted to promote the integration of state-owned and non-state-owned capital, improve corporate governance capabilities and competitiveness, and thus achieve the clearance of zombie firms. Thirdly, cultivate a fair and competitive market environment, and treat state-owned and private capital fairly in market access, administrative approval, licensing, and bidding. At the same time, actively promote the mixed-owned reform in monopolistic industries and the central and western regions, fully unleash positive effects on clearing zombie firms. Fourthly, the conclusion of this article indicates that improving the investment efficiency of state-owned enterprises helps to prevent and resolve zombie firms. For state-owned enterprises, it is necessary to actively integrate the advantages of state-owned and non-state-owned capital, establish a more specialized investment project management team, improve investment efficiency, and reduce the possibility of becoming a zombie firm.

8. The authors must present the limits of the research and the future research directions.

**Answer: Thank you for your suggestions.** We have added the limits of the research and the future research directions as follows:

Although this article provides a comprehensive examination of the relationship between mixed ownership reform and zombie firms, there are still some limitations and directions for further exploration. Firstly, it's important to acknowledge that this article's focus on state-owned enterprises as research samples may introduce bias due to the exclusion of non-listed state-owned enterprises. This limitation could influence regression results and potentially skew conclusions. Additionally, the absence of consideration for the impact of COVID-19 on zombie firms suggests a potential subjectivity in the research findings. Therefore, we encourage future studies to broaden their scope by incorporating data from both developing and developed economies over longer temporal and spatial spans to provide a more comprehensive analysis. Secondly, while the mixed ownership reform entails the integration of state-owned and non-state-owned capital, future research could explore the effects of state-owned capital's involvement in the corporate governance of private enterprises, particularly in addressing issues related to private zombie firms. Lastly, bankruptcy represents a significant mechanism for clearing out zombie firms. Further research could delve into strategies for mitigating the employment and social stability challenges that accompany the bankruptcy of zombie firms.
